# Semi-quantitative assessment of environmental tobacco smoke exposure and its association with the development of oral squamous cell carcinoma: A pilot study

**DOI:** 10.18332/tid/159378

**Published:** 2023-02-28

**Authors:** Susanne Wolfer, Henning Schliephake, Thomas Asendorf, Antonie Spillner, Philipp Kauffmann

**Affiliations:** 1 Department of Oral and Maxillofacial Surgery, University Medical Center Göttingen, Göttingen, Germany; 2 Department of Medical Statistics, University Medical Center Göttingen, Göttingen, Germany

**Keywords:** environmental tobacco smoke, oral squamous cell carcinoma, score

## Abstract

**INTRODUCTION:**

Two known major risk factors for oral squamous cell carcinoma are smoking and alcohol consumption. Environmental tobacco smoke (also known as secondhand smoke) has been proven to be associated with the occurrence of lung and breast carcinoma. This study aimed to assess exposure to environmental tobacco smoke and its association with the development of oral squamous cell carcinomas.

**METHODS:**

Using a standardized questionnaire, 165 cases and 167 controls were asked about their demographic data and risk behaviors, including environmental tobacco smoke exposure. An environmental tobacco smoke score (ETS-score) was developed to semi-quantitatively record the previous exposure to ETS. Statistical analyses were performed with χ^2^ test or Fishers exact test, and with ANOVA or Welch’s t-test as appropriate. An analysis was done using multiple logistic regression.

**RESULTS:**

Cases had a significantly increased previous exposure to environmental tobacco smoke compared to the controls (ETS-score: 36.69 ± 26.34 vs 13.92 ± 12.44; p<0.0001). Comparing only the groups without additional active risk factors, exposure to environmental tobacco smoke was associated with a more than threefold higher likelihood of oral squamous cell carcinoma (OR=3.47; 95% CI: 1.31–10.55). Statistically significant differences in ETS-score were found for different tumor locations (p=0.0012) and different histopathological gradings (p=0.0399). A multiple logistic regression analysis confirmed exposure to environmental tobacco smoke as an independent risk factor for the development of oral squamous cell carcinomas (p<0.0001).

**CONCLUSIONS:**

Environmental tobacco smoke is an important but yet underestimated risk factor for the development of oral squamous cell carcinomas. Further studies are needed to confirm the results, including the usefulness of the developed environmental tobacco smoke score for exposure.

## INTRODUCTION

Lip and oral squamous cell carcinoma (OSCC) are widespread malignancies worldwide, causing many deaths (approximately 170000 annually). Two of the known major risk factors are smoking and alcohol consumption^1,2^. However, many patients with OSCC have shown no risk behavior^3-6^. Environmental tobacco smoke (ETS) (also known as secondhand smoke) has been considered a health risk factor for a number of years^7^. Efforts are being made worldwide to adequately prevent secondhand smoking, for example, through non-smoker protection laws^8^. ETS has been proven to be associated with lung and breast carcinoma^9,10^. Many other diseases, such as diabetes mellitus and cardiovascular diseases, are thought to be associated with secondhand smoke^7,11,12^. There are only a few reports that have examined the impact of ETS on the development of OSCC^13,14^.

The recording of active smoke exposure using pack-years is routinely noted in clinical practice today. However, it is difficult to quantify the exposure to ETS specifically for each individual. In general, exposure to ETS is rarely or not recorded, and only categorically evaluated^13-15^. Quantitative laboratory tests are available, but these can only record the current exposure to active tobacco use or the actual exposure to passive smoking. These tests do not appear to be suitable for everyday clinical routine and for assessing long-term ETS exposure^16-18^.

Passive smoke exposure is usually recorded in case-control studies^7^. The evaluation is carried out with categorical values by determining an odds ratio, which cannot consider individual values. Therefore, a comprehensive overview of the exposure and a comparison of different groups can be difficult. With numerical values, it would be possible to classify the exposure to passive smoking more precisely. An investigation of the influence of ETS as a possible cause of oral squamous cell carcinoma is particularly interesting in the patient group of non-smokers and non-drinkers.

This study aimed to establish an ETS-score for the semi-quantitative assessment of exposure to ETS and the possible association of ETS-score levels with OSCC.

## METHODS

The study was designed as a case-control study. Patients with a history of OSCC were recruited as cases from the regular tumor follow-up between February 2020 to June 2021, using the following inclusion criteria: 1) age ≥18 years; 2) histologically confirmed OSCC; 3) tumor location of the oral tongue as the anterior two-thirds of the tongue, gingiva of the upper jaw, gingiva of the lower jaw, floor of the mouth, palate, buccal mucosa; 4) no immunosuppression; 5) no other malignancy; and 6) capable of giving informed consent and not supervised. Exclusion criteria were: 1) age <18 years; 2) no histologically confirmed OSCC; 3) hypo-, naso- and oropharyngeal location, tumor location outside the oral cavity; 4) immunosuppression; 5) other history of malignancy; and 6) not able to give consent, supervised. Age- and sex-matched inpatients and outpatients without a history of OSCC, aged ≥18 years, without immunosuppression or malignancy, and capable of giving informed consent, served as case controls. The clinical data for the cases were extracted from the clinical records, including the date of diagnosis, location of the OSCC, histology with tumor size, lymph node and metastatic (TNM) stage, residual tumor status (R status), histological tumor grading, nodal extracapsular spread, the incidence of recurrence (census 31 January 2022). A total of 165 cases and 167 controls were included in this study. Details regarding age and sex distribution, among other variables such as marital status and risk behavior, are given in Table 1 and show that there were no significant differences between cases and controls.

**Table 1:** Characteristics of cases and controls, February 2020 to June 2021

*Characteristics*	*Cases N=165 n (%)*	*Controls N=167 n (%)*	*p*
**Age** (years), mean ± std	62.16 ± 11.44	63.99 ± 10.94	0.1327
**Sex**	n=165	n=167	0.0786
Male	93 (56.36)	78 (46.71)	
Female	72 (43.64)	89 (53.29)	
**Marital status**	n=164	n=167	0.0583
Single	26 (15.85)	14 (8.38)	
Married	109 (66.46)	126 (75.45)	
Divorced	12 (7.32)	6 (3.59)	
Widowed	14 (8.54)	13 (7.78)	
Permanent partner	3 (1.83)	8 (4.79)	
**Risk behavior**	n=164	n=167	<0.0001
NSND	37 (22.42)	60 (35.93)	
SND and NSD	67 (40.60)	86 (51.50)	
SD	61 (36.97)	21 (12.57)	
**Pack-years,**	n=154	n=159	<0.0001
mean ± std	24.95 ± 26.97	7.78 ± 11.75	
**ETS history**, n	n=163	n=167	0.0011*
ETS+	145	124	
ETS-	18	43	
**ETS-score,**	n=163	n=167	<0.0001
mean ± std	36.69 ± 26.34	13.92 ± 12.44	

NSND: non-smoker–non-drinker. SND: smoker–non-drinker. NSD: non-smoker–drinker. SD: smoker–drinker. ETS: environmental tobacco smoke. std: standard deviation.

* OR=2.77 (95% CI: 1.54–5.26).

### Exposure to ETS

After the patient’s written consent to participate in this one-time survey, a standardized questionnaire was completed by the participants with the supervision of one single trained interviewer to exclude an inter-interviewer variation. Data on demographic parameters such as age, sex, and relationship status at diagnosis were recorded. Exposure to ETS was specifically asked for and registered separately for ETS at work and ETS at home. Any exposure to tobacco smoke that was not caused by the participant’s smoking behavior but by other people at work or at home was classified as environmental tobacco smoke exposure. To explore ETS exposure at home, the smoking habits of spouses or life partners and parents were asked and whether this had resulted in participants’ exposure to ETS. Scores were attributed as follows: 1) duration of ETS in years, and 2) frequency of exposure to ETS recorded as ETS rate (never=0, occasionally=0.5, constantly=1). Occasionally means now and then, irregularly; constantly means every day, regularly. Data were used to calculate a simple and easy to use composite score that reflected exposure to environmental tobacco smoke using the following formula:

*ETS score = (ETS years at home × ETS rate at home) + (ETS years at work × ETS rate at work)*

For example: constant ETS exposure at home for 10 years and an occasional ETS exposure at work for 20 years results in an ETS-score of (10×1) + (20×0.5) = 20. A high score represents high exposure to ETS, and vice versa.

### Risk behavior

Current and previous risk behavior (tobacco smoking and alcohol consumption) and information about the amount and duration of smoking and alcohol consumption was recorded, as well as possible periods of cessation. The intensity of active smoking was expressed in pack-years [cigarette packs per day (20 cigarettes/pack) × years smoked). Patients were divided into NSND (non-smoker–non-drinker, without any risk behavior), SND (smoker and non-drinker), NSD (non-smoker and drinker) with one risk behavior, and SD (smoker and drinker) as participants with two risk behaviors. Participants who had never smoked were considered non-smokers, and participants who regularly consumed alcohol every day were considered drinkers.

### Statistical analysis

Baseline characteristics were compared by Welch’s t-test/ANOVA or Fisher’s exact test/ test, as appropriate. Group comparisons of the ETS-score were performed using either Welch’s t-test or ANOVA for more than two groups. Mean ± standard deviation (std) values are reported for numerical variables. Risk differences between cases and controls depending on exposure to ETS were assessed by odds ratio, and in a multiple logistic regression analysis for covariates age, sex, pack-years and smoker/drinker category using generalized linear models. The two-sided significance level was set to 5% for all statistical tests and 95% confidence intervals were reported for values of interest.

## RESULTS

**Figure 1:**
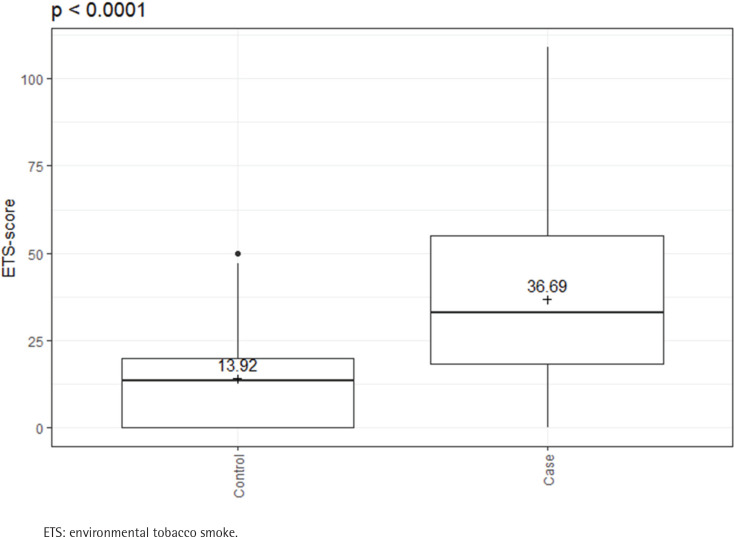
ETS-scores of cases (N=163) and controls (N=167); Cases have significantly higher ETS exposure than controls, February 2020 to June 2021

There were clear differences in risk behavior and in ETS exposure between the cases and controls. Cases were more frequent SDs (36.97% vs 12.57%; p<0.0001) and had a higher number of pack-years (24.95 ± 26.97 vs 7.78 ± 11.75; p<0.0001). The ETS-score shows a significantly increased exposure in the cases compared to the controls (36.69 ± 26.34 vs 13.92 ± 12.44; p<0.0001) (Table 1 and Figure 1).

The unadjusted exposure to ETS at home and at work (in years) was higher in the cases than in the controls (23.15 ± 21.15 vs 11.56 ± 13.72 at home; 16.67 ± 17.29 vs 6.44 ± 11.63 at work; p<0.0001). Also, significantly more cases reported exposure to ETS at both places, i.e. home and work (39.63% vs 14.97%; p<0.0001). The unadjusted odds ratio (OR) of ETS (no exposure vs exposure) increased from 1.95 (95% CI: 1.23–3.12) at home and 2.67 (95% CI: 1.71–4.21) at work to as much as 3.70 (95% CI: 2.20–6.38) with exposure to ETS at both places (Table 2).

**Table 2:** Distribution of the exposure to environmental tobacco smoke (ETS) for cases and controls; Cases had a higher exposure to ETS at home, at work and at both places, compared to controls, February 2020 to June 2021

*Exposure*	*Cases N=164 n (%)*	*Controls N=167 n (%)*	*p*	*OR (95% CI)*
**Exposure to ETS**				
**At home**	n=164	n=167	0.0039	1.95 (1.23–3.12)
Yes	119 (72.56)	96 (57.49)		
No	45 (27.43)	71 (42.51)		
**At work**	n= 162	n=167	<0.0001	2.67 (1.71–4.21)
Yes	91 (55.49)	53 (31.74)		
No	73 (44.51)	114 (68.26)		
**At home and work**	n=162	n=167	<0.0001	3.7 (2.20–6.38)
Yes	65 (39.63)	25 (14.97)		
No	99 (60.37)	142 (85.03)		
**ETS at home**	n=164	n=167	<0.0001	
Never	45 (27.16)	71 (42.51)		
Occasionally	16 (9.25)	36 (21.56)		
Constantly	103 (63.58)	60 (35.93)		
**ETS at home** (years), mean ± std	23.15 ± 21.15	11.56 ± 13.72	<0.0001	
**ETS at work**	n=164	n=167	<0.0001	
Never	73 (44.51)	114 (68.26)		
Occasionally	16 (9.76)	20 (11.98)		
Constantly	75 (45.73)	33 (19.76)		
**ETS at work** (years), mean ± std	16.67 ± 17.29	6.44 ± 11.63	<0.0001	
**ETS from smoking partner**	n=146	n=166	0.0143	
Never	104 (71.23)	138 (83.13)		
Occasionally	7 (4.85)	10 (6.02)		
Constantly	35 (23.97)	18 (10.84)		

**Figure 2:**
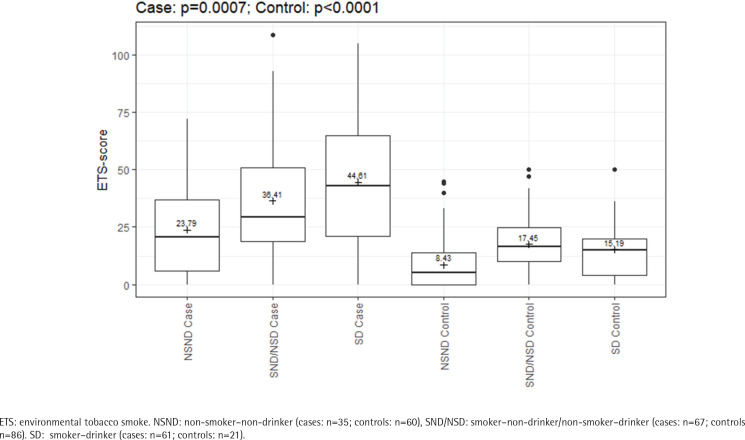
ETS-score regarding the different risk behavior; With increasing risk behavior there is an increasing ETS exposure in the cases (N=163) compared to the controls (N=167), February 2020 to June 2021

**Figure 3:**
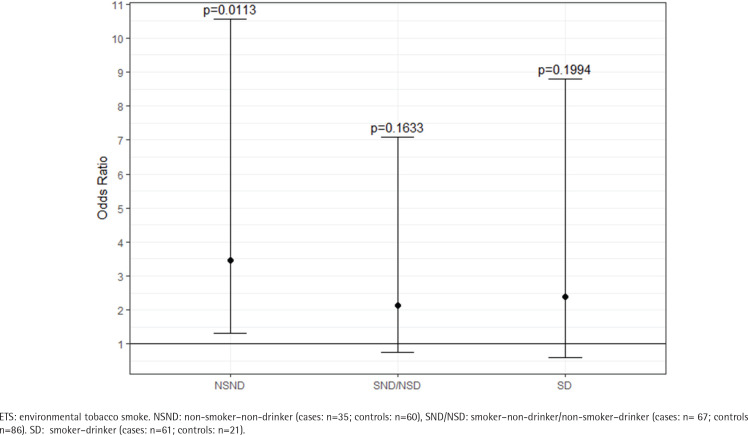
Odds ratios for the different risk behavior groups; note the high odds ratio for the NSND group compared to the other risk groups (cases: N=163; controls: N=167), February 2020 to June 2021

ETS exposure was also significantly different between cases and controls within the various risk groups, which is shown in detail in Supplementary file Table S1. The group of NSNDs recorded less exposure to ETS than participants with positive risk behavior. In the cases, there was a clear increase in the ETS-score with increasing risk behavior (23.79 vs 36.41 vs 44.61; p<0.0067) (Figure 2). Among the NSND cases, ETS exposure was associated with three-fold odds of developing an OSCC than among the NSND controls (OR=3.47; 95% CI: 1.31–10.55) (Figure 3 and Supplementary file Table S2).

**Figure 4:**
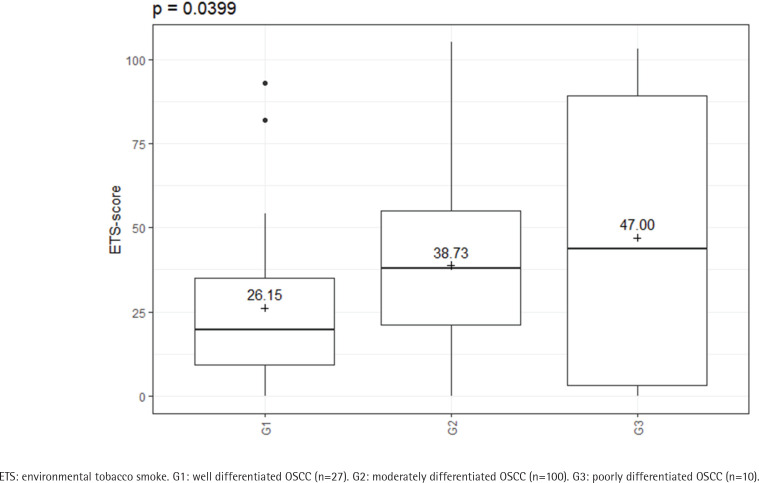
ETS-score regarding the histopathological grading (cases: N=137); There are increasing ETS-scores for increasing grading classifications, February 2020 to June 2021

The amount of ETS experienced by the cases also appeared to have an influence on the tumor characteristics. In this study, different ETS-scores were found for the different tumor locations (p=0.0012) (Supplementary file Figure S1). A significant difference was also found in the histopathological grading (p=0.0399) (Figure 4). The amount of ETS appeared to have no effect on the T and N stages, and the recurrence (p>0.05). Likewise, no difference could be determined as to whether the patients had a nodal status with or without extracapsular spread (p>0.05) (Supplementary file Table S3).

The multiple logistic regression analysis confirmed the independent influence of the ETS on the development of OSCC (p<0.0001). With an average marginal effect of 0.01, it was approximately shown that with an increase of the ETS-score by 1 point, the risk of OSCC increases by 1% in our study (Table 3).

**Table 3:** Average marginal effects (AME) for the occurrence of an OSCC; AME are calculated by multiple logistic regression for age, gender, pack-years, ETS-score, and smoker/drinker risk groups (cases: N=153; controls: N=159), February 2020 to June 2021

*Variable*	AME*	*95% CI*	*p*
Age	-0.0026	-0.0067 – 0.0016	0.2241
Gender	0.0318	-0.0646 – 0.1282	0.5182
Pack-years	0.0064	0.0027 – 0.0100	0.0006
ETS-score	0.0102	0.0079 – 0.0125	<0.0001
NSD/SND risk group	-0.1721	-0.2795 – -0.0646	0.0017
SD risk group	-0.0393	-0.2079 – -0.1294	0.6482

* With an average AME of 0.01, it is shown that with an increase of the ETS-score by 1 point, the hazard to develop an OSCC increases by 1%. ETS: environmental tobacco smoke.

## DISCUSSION

In our study, the cases had a significantly increased exposure to ETS compared to the controls. Comparing just the groups without additional active risk factors (NSND groups), a history of ETS exposure was three times more likely in the cases than in the controls.

ETS has been reported as an independent risk factor for the development of OSCC in Chinese women^13^, where a multiplicative interaction between passive smoking and exposure to cooking oil fumes was found, with OR values ranging 1.52–2.38, in line with our results. He et al.^13^ had recorded the ETS categorically. With the ETS-score it is possible to record a numerical value for each individual. Other case-control studies report a dose-response relationship for the degree of ETS exposure and describe secondhand smoke as an independent predictor of recurrence and survival in patients with head and neck cancer^14,15^. Zhang et al.^14^ found an increased OR for heavy ETS exposure (exposure at home and at work) in comparison to moderate ETS exposure (exposure only at home or at work) in their study group. This was also true when only NSND patients were analyzed. This is very much in line with our results, where the OR is also higher with exposure at both places. In contrast to the results of Idris et al.^15^, we could not determine any difference in the occurrence of recurrences in our patients with and without exposure to ETS. However, Zhang et al.^14^ and Idris et al.^15^ had evaluated patients with head and neck squamous cell carcinoma and in one study, only about half of the participants were patients with OSCC, and in the other study, no information was given on the percentage of patients with oral cancer. Again, both use a categorical assessment of the ETS exposure, and levels were graded as no, moderate, or high, exposure with ETS occurrence at home, at work, or at both. Information on the duration of exposure was not recorded. However, we believe it matters how long (in years) the exposure lasted. Therefore, we included the exposure time in years in the calculation of the ETS-score. Also, it makes a difference whether the exposure to ETS occurred constantly or occasionally. Therefore, we developed the ETS-score based on these three studies^13-15^, taking into account the place of exposure, the duration (in years), and the exposure rate (none, occasionally, constantly). In this way, the ETS exposure is to be recorded as precisely as possible with the ETS-score, and at the same time, the exposure to ETS can be represented numerically. The ETS score is certainly an approximation, but as a numerical value, it is better to use for statistical evaluations. Furthermore, the amount of exposure is clearly visible with the ETS-score, which allows the sorting of low after high as a type of quantitative measure. The increasing ETS-score with increasing risk behavior of the patients in our study shows that a good differentiation is possible. Zhang et al.^14^ also described the influence of passive smoking in active smokers, since they probably spend more time with other smokers and inhaled the side stream smoke of the other smokers in addition to mainstream smoke. They describe an elevated prevalence of ETS with an increased number of pack-years. Also, Dahlstrom et al.^5^ had described an increase of ETS exposure from NSND to ever smokers and ever drinkers, which was reported in percentages at home or at work, or at both. So we can confirm these results. However, we can additionally represent this as a visible and comparable value with the ETS-score.

For our study group, we could show with the multiple logistic regression analysis that ETS was significantly higher in the cases than in the controls, indicating that ETS may be an independent risk factor for the development of OSCC. Furthermore, we found an increased risk by 1% with an increasing ETS-score by 1 point. This, to our knowledge, has never been reported in the literature, and shows the good functionality of the developed ETS-score in evaluating the exposure to ETS.

Moyses et al.^19^ did not regard ETS as a major risk factor for OSCC^1^, mentioning the difficulty in measurement of lifetime ETS load and that data about the ETS exposure are not routinely recorded in clinical practice. With the ETS score, this is possible and the required data can be collected easily and clinically practicably, comparable to collecting the data for the calculation of the pack-years.

Different lifestyle risk factors for oral cancer were discussed by Petti^20^ but ETS was not included in that extensive review. NSNDs are a particularly important patient group when considering the effects of ETS exposure. A significant proportion of patients with OSCC are NSND, with the majority women^3-5,21,22^. It was discussed, that NSND differ in carcinogenesis mechanisms typically associated with smoking, and that there are other genetic alterations or not yet investigated environmental causes involved^21^. In this study, we investigated the ETS exposure as an environmental cause. The proportion of women in the NSND cases is high (86.5%; 32/37). When comparing the ETS-scores, however, no difference in sex could be determined in any of the risk groups or in the cases and controls. This means that exposure to ETS is similar in both sexes in our study groups. This is in line with the results by Zhang et al.^14^ who also saw no difference in sex and age. Other studies report that there is a high level of secondhand smoke exposure in women due to smoking spouses^5,9,23^. We also found a significantly higher exposure to ETS from the spouse among the cases compared to the controls, regardless of gender and risk profile, and thus, we partially support these statements. Due to the small number in the subgroups, a separate analysis of the sole NSND groups and female participants has not been carried out.

NSND were also particularly considered when examining cases of breast cancer and lung cancer. ETS is described as an important risk factor for lung cancer in female non-smokers^9,24^. Non-smoking women who live with smoking men have a 24% increased risk to develop lung cancer compared to non-smoking women who were not exposed to ETS^24^. As an explanation, the exposure to ETS which contains many carcinogens, was given^9,23^.

It is well known that tobacco smoke causes a variety of cancers. However, it is not only the airways, which represent the direct path of the smoke during active inhalation, that are affected. In addition to lung, laryngeal, pharyngeal and OSCC, carcinomas of the esophagus and also of the liver, bladder, cervix, kidneys and pancreas that are very distant from the respiratory tract have been described as being tobacco-related^20,22,25^. More than 60 carcinogens have been identified within tobacco smoke^20^. The carcinogens of tobacco smoke are thought to cause direct and indirect DNA mutations, and indirect DNA damage which disturbs the cellular processes^22^ and switches tumor suppressor genes or oncogenes, on or off, and related to the development of OSCC^26^. Thus, normal keratinocytes can transform into malignantly growing keratinocytes^26^. Increased DNA adducts were found in smoking patients with OSCC, and protein adduct measurement can distinguish non-smokers exposed to ETS from those not exposed^25^. Furthermore, an animal study analyzed the tongue epithelial response to cigarette smoke exposure, and concluded that cigarette smoke exposure induces the risk of oral cancer development^27^. It is, therefore, conceivable that the carcinogens taken within ETS can also cause OSCC in NSND, for which the mechanisms of formation with different lifestyle factors are still largely unknown, and furthermore, the carcinogens can increase the risk of OSCC in active smokers^15,28^. The ETS-score in our study indicates increased exposure to ETS, associated especially with cancers of the floor of the mouth and lower jaw gingival, which due to the their location can result in an increased accumulation of carcinogens in the saliva-collecting regions. If one compares the ETS-scores of the different risk groups with those of the different locations, there may also be a connection between the increased risk burden in SD and floor of the mouth, and in the NSND and the tongue. It has been described that floor of the mouth carcinoma is more likely to occur in smokers, including women who smoke, and tongue carcinoma more in non-smokers^5,28^. Recently, different studies report that tobacco smoke alters the structure of the oral microbiome and shifts it to dysbiosis. The composition of the oral bacterial and fungal species in the saliva changes, and therefore changes occur which alter the cell and tissue re-modeling, the suppression of apoptosis, and the secretion of carcinogenic toxins^29-31^. However, the reason for the emergence of OSCC in NSNDs is still largely unknown. ETS exposure could be one possible explanation of what is likely a multifactorial and complex process.

### Limitations

A statistical evaluation of the ETS-score and the localization divided according to the various risk groups was not carried out in this study due to the small number within the subgroups. This requires further studies with a much larger number of participants. A further limitation of this study is that a case-control study cannot really demonstrate cause and effect. For that, large cohort studies are required. But even with cohort studies, the amount of ETS exposure is very difficult to measure and must last for many years. Case-control studies are very common and widely used because of their practicability to clarify these questions about the influence of ETS in the development of different cancers^9,10^. Another limitation of the study is that we do not record the ETS exposure from free-time activities, and from public places, for example. This ETS exposure is certainly also a part of the ETS load that should not be underestimated. Due to this even more irregular exposure, it is even more difficult to capture, both categorically and numerically. With the ETS-score, however, at least the exposure occurring at the main sites (home and workplace) can be sufficiently quantified. It must also be mentioned that the ETS exposure was recorded by a questionnaire survey, which is based on recall of past circumstances of the participants, and has the potential to be incorrect. In the literature, the possible misclassification of the smoking status by questionnaires as a possible bias is discussed and the assessment of the tobacco smoke exposure by objective biomarkers like cotinine is mentioned^17,32^. Urinary cotinine as a major metabolite of nicotine that can be used as a short-term biomarker with half-life of only 18h, which is reported to have a strong correlation with self-reported exposure to tobacco smoke^17^. But for the assessment of the total exposure to ETS, a period over many years is necessary, therefore the use of the urinary cotinine level is impractical^16^. In the literature, we found that self-reported exposure to ETS is to be regarded as valid both in the short-term and the long-term^14^. Furthermore, the potential bias due to smoker misclassification was assessed as unlikely to be responsible for the increased health risk observed in studies on ETS^17^. We have provided a possible instrument to record numerically ETS exposure of patients. It represents only an approximation, as does the calculation of the pack-years, but the ETS-score differentiates the exposure to ETS well enough, is a numerical value for statistical use, and is easy to obtain for the participants and the investigators. This makes it suitable for everyday clinical use. Further studies with much larger number of participants must are needed to confirm the practicability of the ETS score and to validate the results of this pilot study.

## CONCLUSIONS

ETS is an important, yet underestimated, risk factor for developing oral squamous cell carcinoma. The risk increases with increasing additional risk behavior. The presented ETS-score has shown to be a sensitive measure for semi-quantitative assessment of ETS exposure. Further studies are needed to confirm the results and validate the usefulness of the ETS-score as a numerical measurement of environmental tobacco smoke exposure. This ETS-score could be used as an easy-to-use instrument in everyday clinical practice for all known diseases but also for the detection of other diseases possibly associated with ETS.

## Data Availability

The data supporting this research are available from the authors on reasonable request.
