# Supplementary material for: Semi-quantitative assessment of environmental tobacco smoke exposure and its association with the development of oral squamous cell carcinoma: A pilot study
Source: Tob Induc Dis. 2023 Feb 28;21:32. doi: 10.18332/tid/159378 (PMC9972359; doi:10.18332/tid/159378)
Supplement: Supplementary file 1 [file TID-21-32-s1.pdf]

Supplemental material:

Variable / ETS-score	NSND		SND/NSD		SD		
Case<br>m<br>f	 n=5<br>n= 32	 20.25± 14.24<br>24.23± 20.76	 n= 37<br>n= 30	 35.59± 24.10<br>37.45± 26.76	 n= 51<br>n= 10	 43.89± 29.20<br>48.30± 22.94	
p value m vs f	0.6411		0.77156		0.6042		
Control<br>m<br>f	 n= 22<br>n= 38	 6.136± 10.05<br>9.750±11.16	 n= 40<br>n= 46	 19.84± 13.21<br>15.37± 10.50	 n= 16<br>n= 5	 15.63± 13.86<br>13.80± 14.08	
p value m vs f	0.2038		0.0898		0.8072		
	NSND		SND/NSD		SD		p value<br>NSND vs SND/NSD vs SD
Case<br>Control	n= 37<br>n= 60	23.79± 20.012<br>8.425± 10.82	n= 67<br>n= 86	36.41± 25.12<br>17.45± 11.98	n= 61<br>n= 21	44.61± 28.15<br>15.19± 13.58	0.00067<br><0.0001
p value<br>case vs control	< 0.001		< 0.001		< 0.001		

Supplement Table S1: ETS exposure for different risk groups, measured with the ETS-score (cases n=165, controls n=167), 2021; Please note the increasing ETS exposure with the increasing risk behavior in the case group. For the controls, there are only an increase from NSND to risk positive participants. For cases and controls, there were no differences in gender for all risk groups.
Abbreviations: n = number, NSND = non-smoker-non-drinker, SND&NSD = smoker-non-drinker and non-smoker-drinker, SD = smoker-drinker, ETS = environmental tobacco smoke, m = male, f = female

	NSND		SND/NSD		SD	
	case	control	case	control	case	control
ETS +	30	35	61	73	54	16
ETS -	6	25	5	13	7	5
OR (95% CI)	3.47 (1.31-10.55)		2.13 (0..75-7.09)		2.40 (0.62-8.78)	
p value	0.0113		0.1633		0.1994	

Supplement Table S2: Odds ratios for development of an OSCC with exposition to ETS (cases n=165, controls n=167), 2021; please note the significant Odds ratio for the NSND group.
Abbreviations: NSND = non-smoker-non-drinker, SND&NSD = smoker-non-drinker and non-smoker-drinker, SD = smoker-drinker, ETS = environmental tobacco smoke, OR = odds ratio, CI = 95% confidence interval

variable	Number (%)	ETS-score mean/median (sd)	p value
Location n= 163			0.0012
FOM	45 (27.61)	48.71/46 (27.18)	
Tongue	37 (22.70)	26.88/22 (22.38)	
Alveolar rim upper jaw	11 (6.74)	24.86/20 (17.87)	
Alveolar rim lower jaw	43 (26.38)	40.31/42.25 (26.78)	
Palate	14 (8.59)	28.79/18 (29.97)	
Buccal mucosa	13 (7.98)	30.42/31.5 (19.13)	
T stage n= 152			0.3530
T1	66 (43.42)	37.65/35 (22.51)	
T2	52 (34.21)	34.88/32 (26.41)	
T3	16 (10.53)	31.78/26 (31.81)	
T4	18 (11.84)	46.06/46.5 (25.53)	
N stage n= 152			0.9509
N0	106 (69.74)	37.12/35 (25.94)	
N1	21 (13.81)	35.17/37 (25.98)	
N≥2	25 (16.44)	36.56/30 (26.35)	
ECS n= 149			0.1437
ECS 0	135 (90.60)	38.17/37 (26.51)	
ECS 1	14 (9.40)	27.50/22.5 (17.82)	
R stage n= 147			0.8161
R0	144 (97.96)	36.79/35 (26.01)	
R1	3 (2.04)	40.33/24 (30.04)	
R2	0	0	
Grading n= 138			0.0399
G1	26 (18.84)	26.15/19.5 (23.59)	
G2	102 (73.91)	38.73/38 (24.01)	
G3	10 (7.25)	47.00/43.75 (43.15)	
Recurrence n=160			0.8915
Yes	42 (26.25)	36.35/27.25 (27.81)	
no	118 (73.75)	37.00/35.00 (26.01)	

Supplement Table S3: ETS exposure for the different histopathological variables, measured with the ETS-score (cases n=163), 2021; There were significant differences for the different tumor locations and for the different histopathological gradings. No significances were found for T-, N- and R-stage, for ECS and recurrence.

Abbreviations: n = number, ETS = environmental tobacco smoke, FOM = floor of the mouth, T-stage = tumor stage, N-stage = nodal stage, ECS = extracapsular spread, R-stage = stage of resection margin, G1 = well differentiated tumor, G2 = moderate differentiated tumor, G3 = poor differentiated tumor, CI = 95% confidence interval

Figure legend:

Figure S1: ETS-score for the various tumor locations, n=161, 2021; There are different exposures to ETS for the different tumor locations. Please note the high ETS-scores for FOM and for the alveolar rim of the lower jaw in comparison to the tongue, the alveolar rim of the upper jaw and the palate. Abbreviation: ETS = environmental tobacco smoke, FOM = floor of the mouth

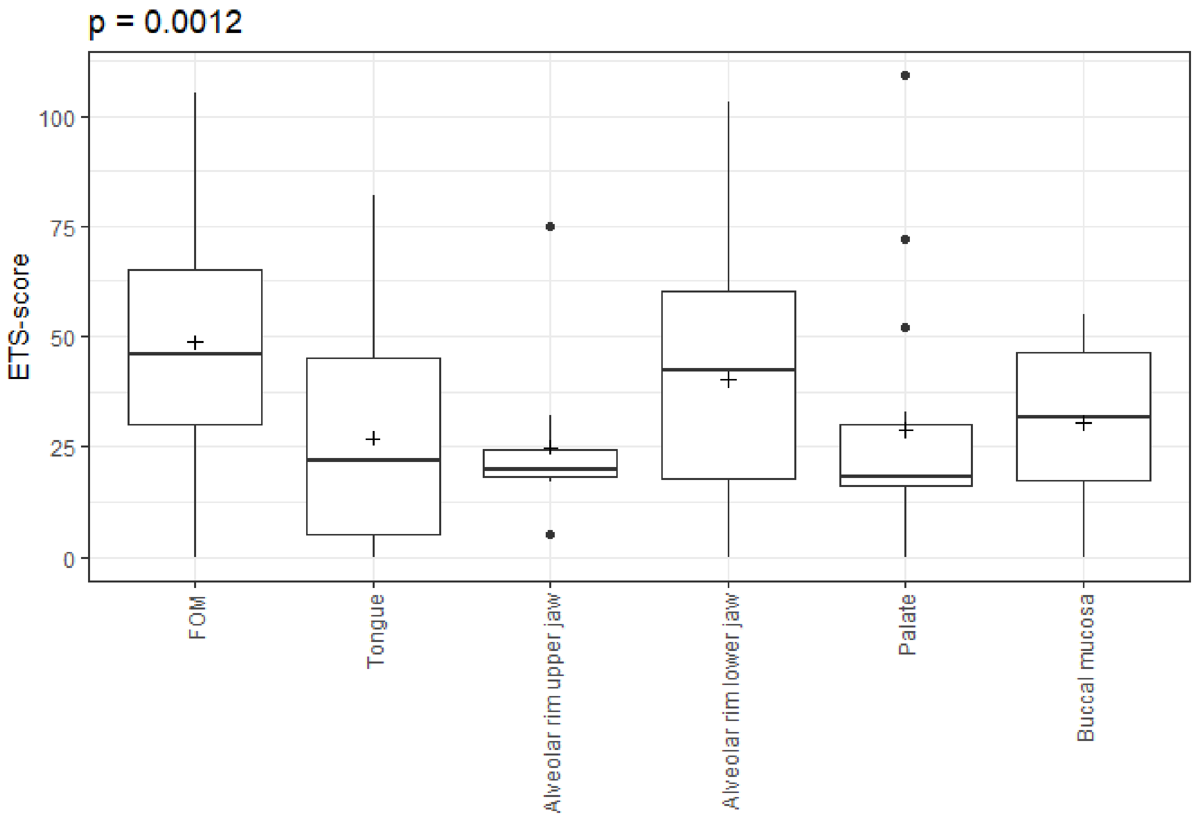

© 2023 Wolfer S. et al.
